# Accelerating imaging: deep learning for enhanced ^123^I-ioflupane SPECT efficiency

**DOI:** 10.1007/s11604-025-01933-z

**Published:** 2025-12-18

**Authors:** Yoshinobu Ishiwata, Keiichi Horie, Kazuhiro Aritome, Ryo Aoki, Hitoshi Iizuka, Shinjiro Aso, Yuka Takeuchi, Yuka Misumi, Akira Haga, Shingo Kato, Tsuneo Yamashiro, Shoko Takano, Daisuke Utsunomiya

**Affiliations:** 1 Department of Radiology, Yokohama City University Hospital, 3-9 Fukuura, Kanazawa-Ward, Yokohama, 2360004 Japan; 2 Department of Data Science, Yokohama City University, 2-22 Seto, Kanazawa-Ward, Yokohama, 2360027 Japan; 3 Department of Radiology, Yokohama City University Medical Center, 4-57 Urafune, Minami-Ward, Yokohama, 2320024 Japan; 4 Department of Nuclear Medicine, Yokohama City University Hospital, 3-9 Fukuura, Kanazawa-Ward, Yokohama, 2360004 Japan

**Keywords:** Dopamine transporter imaging, Parkinsonism, Single-photon emission computed tomography (SPECT), Deep learning, Scan time reduction

## Abstract

**Background:**

Conventional ^123^I-ioflupane dopamine-transporter SPECT requires 25–40 min of acquisition, causing patient discomfort and limiting throughput. This study assessed whether deep-learning (DL) reconstruction can yield diagnostic-quality images from a 5-min scan.

**Methods:**

We retrospectively analysed 207 studies (1035 slices) obtained between April 2018 and June 2020. After cropping to 64 × 64 striatal regions, 600, 185 and 250 images from 120, 37 and 50 patients were used for training, validation and testing. Six convolutional architectures—U-Net (one–five depths), V-Net, U-Net +  + , R2U-Net, Attention U-Net and TransUNet—were trained to translate 5-min into virtual 25-min images. Image quality was assessed with peak signal-to-noise ratio (PSNR) and structural similarity index (SSIM), analysed by Friedman and Dunn–Holm tests. A blinded reader study involved three nuclear medicine physicians grading 50 cases (100 striata) on a four-point scale; agreement with the 25-min consensus was measured by weighted κ and intra-/inter-observer intraclass correlation coefficients (ICC).

**Results:**

All DL reconstructions significantly outperformed raw 5-min images in PSNR and SSIM (p < 0.01). The four-layer U-Net achieved the highest quality (PSNR 32.7 ± 1.7 dB, SSIM 0.842 ± 0.069), ≈1.8 dB and 0.13 higher than baseline, and statistically indistinguishable from 25-min images (*p* > 0.05). Reader concordance improved from fair with baseline (κ = 0.29–0.41) to substantial with the four-layer U-Net (κ = 0.62–0.70); intra-reader ICC was 0.84–0.93 and inter-reader ICC 0.73–0.75.

**Conclusions:**

A compact four-layer U-Net restores diagnostic fidelity to 5-min ^123^I-ioflupane SPECT, enabling an 80% reduction in scan time without loss of quantitative metrics or interpretability. DL-accelerated protocols may enhance comfort, reduce motion artefacts and increase throughput, warranting prospective multicentre validation.

**Supplementary Information:**

The online version contains supplementary material available at 10.1007/s11604-025-01933-z.

## Introduction

^123^I-Ioflupane, a radiopharmaceutical crucial for dopamine transporter imaging, plays a significant role in the diagnosis of Parkinson’s disease and dementia [1, 2]. The annual number of ^123^I-Ioflupane imaging procedures has been steadily increasing, and this trend is expected to continue, reflecting its indispensable role in clinical practice. However, acquiring diagnostically sufficient images with an adequate signal-to-noise ratio (SNR) typically requires a prolonged acquisition time of 30 to 40 min [3, 4]. This extended imaging duration imposes several challenges. Patients are required to maintain the same posture for an extended period, often resulting in significant physical and psychological distress. Furthermore, the prolonged acquisition time increases the workload for co-medical staff, reduces the efficient utilization of costly and limited imaging equipment, and raises concerns about examination efficiency and cost-effectiveness. These limitations highlight the urgent need for strategies to reduce the acquisition time while maintaining diagnostic accuracy. Recent advancements in artificial intelligence, particularly deep learning, have demonstrated significant potential in improving image quality and reducing acquisition times across various imaging modalities. Notable progress has been achieved in MRI, where deep learning has been successfully applied to shorten acquisition times while preserving diagnostic quality [5, 6]. In contrast, similar developments in nuclear medicine have been comparatively limited, with most efforts focusing on PET or SPECT imaging. Deep learning-based studies of image quality improvement typically rely on paired datasets consisting of noisy images from reduced radiopharmaceutical doses or short-time imaging and noisy standard images. By training models on these datasets, it is possible to generate virtual standard images that can be used to reduce radiopharmaceutical dosage or shorten imaging time without compromising diagnostic utility. Examples in nuclear medicine include the generation of myocardial blood flow scintigraphy images from low dose or short time imaging images and the application of Deep Denoising Super-Resolution Convolutional Neural Network (DDSRCNN) for noise reduction in low count bone scintigraphy images [7–9]. Furthermore, deep learning-based noise reduction techniques have been widely explored for PET imaging [10, 11]. A recent study compared the performance of various convolutional neural network (CNN) architectures for noise reduction [12], while another introduced a noise reduction framework using a diffusion model, a type of deep generative model [13]. Despite these promising developments, the application of such techniques to ^123^I-Ioflupane imaging remains unexplored. Given the unique characteristics of ^123^I-Ioflupane, including its stable biodistribution in the brain post-intravenous administration, it represents an ideal candidate for the application of deep learning-based image enhancement. This study aims to address the challenges posed by the prolonged acquisition time of ^123^I-Ioflupane imaging. Specifically, we propose a deep learning-based approach to shorten imaging time while maintaining diagnostic quality. By evaluating the concordance between the original long-acquisition images and those generated from short-acquisition data, we aim to establish the clinical utility of this novel approach. This investigation represents a pioneering effort in applying artificial intelligence to optimize ^123^I-Ioflupane imaging, with the potential to improve patient comfort, operational efficiency, and healthcare resource utilization.

## Materials and methods

### Data acquisition

**Fig. 1:**
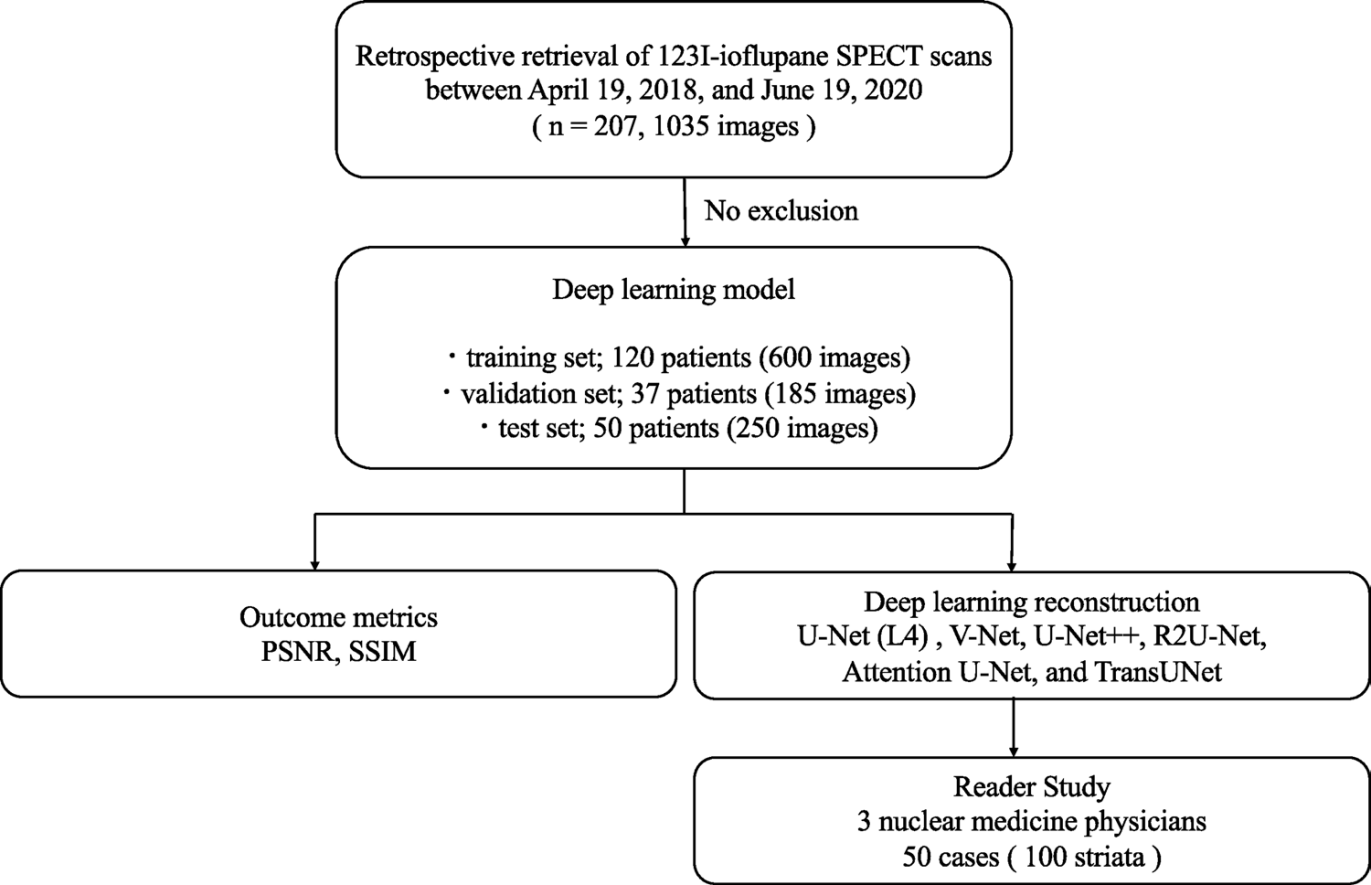
Study flow chart. PSNR: Peak signal-to-noise ratio, SSIM: Structural similarity index measure, SPECT: Single-photon emission computed tomography, U-Net: U-Net (encoder–decoder CNN), U-Net +  + : Nested U-Net, V-Net: V-Net (3-D CNN), R2U-Net: Recurrent residual U-Net, Attention U-Net: Attention-gated U-Net, TransUNet: Transformer-based U-Net

This retrospective-single center study was approved by the Ethical Review Committee of Yokohama City University Hospital (Approval Number: B201200072; date, 19/01/2019) and conducted in accordance with the Declaration of Helsinki. The informed consent was waived due to the retrospective nature of the study. This study includes 207 patients who underwent 123I-ioflupane imaging at Yokohama City University Hospital between April 19, 2018, and June 19, 2020. There were no restrictions on age or sex, and no exclusion criteria were applied. The patient cohort consisted of 104 males, 102 females, and 1 unknown sex, with a mean age of 71.50 ± 10.05 years. The 207 patients included a wide range of clinical diagnoses: 111 (53.6%) with Parkinson’s disease (PD), 15 (7.2%) with Dementia with Lewy Bodies (DLB), 10 (4.8%) with Progressive Supranuclear Palsy (PSP), 17 (8.2%) with Essential Tremor (ET), and 54 (26.1%) with other conditions (e.g., drug-induced parkinsonism, Alzheimer’s disease, or unknown). The diagnostic breakdown of the training, validation, and test datasets is provided in Table 1. For each patient, anonymized 123I-ioflupane images were acquired and output with acquisition times of 5 (short time image) and 25 min (original image) (Fig. 1). Scatter correction and CT-based attenuation correction were applied to all output images. The number of image slices per case ranged from 59 to 83, with a total of 14,530 images across all acquisition times. ^123^I-FP-CIT scans were taken in the supine position 3 to 4 h after the intravenous administration of 185 MBq of FP-CIT to the patient using a SPECT/CT scanner with 16 detector rows (Symbia T2, Siemens Healthcare, Germany) equipped with a dual-detector system. Low- and medium-energy general-purpose (LMEGP) collimators were used. SPECT data acquisition was carried out over an angular range of 180˚ (4˚ steps at 45 frames). The scan was performed in 5 cycles, with each cycle lasting approximately 5 min, resulting in a total acquisition time of approximately 25 min. This protocol allowed for the generation of two datasets: the original image (reconstructed from the full 25-min data) and the short-time image (reconstructed from only the first 5-min cycle data). Image reconstruction was performed using a SYNGOP workstation (Siemens Healthcare, Forchheim, Germany).

**Table 1:** Clinical diagnoses of the patient cohort

Diagnosis	Frequency (n)	Diagnosis	Frequency (n)
Parkinson’s diseases	57	Major depressive disorder	2
Progressive Supranuclear Palsy	29	Sequelae of cerebrovascular disease	2
Multiple system atrophy	18	Vascular Parkinsonism	2
Dementia with lewy bodies	16	behavioral variant frontotempora] dementia	1
Drug-induced parkinsonism	10	Beta-propeller protein-associated neurodegeneration	1
Essential tremor	10	Cerebellar ataxia, neuropathy, and vestibular areflexia syndrome	1
Alzheimer’s disease	9	Delusional disorder	1
Corticobasal syndrome	9	Disuse syndrome	1
Age-related changes	6	Lumbar spinal stenosis	1
Undiagnosed	6	Parkinson’s disea&e dementia	1
Amyotrophic lateral sclerosis	5	Primary lateral sclerosis	1
Spinocerebellar ataxia	5	Progressive Nontluent Aphasia	1
Normal pressure hydrocephalus	4	Pure autonomic failure	1
Dropped head syndrome	3	Somatic symptom disorder	1
Epilepsy	2	Van da Knaap	1

### Image preprocessing

To isolate data relevant to the striatum, we calculated the maximum count number, normalized for each case, for all slice images obtained during the 25-min acquisition, as described in a previous report [14]. The slice with the highest maximum count number was considered optimal for visualizing the striatum. Based on this, five slices were selected for analysis: the slice with the highest count and the two slices immediately preceding and following it. The same slice numbers were extracted for the 5-min acquisition images. All images used in the experiment were normalized. Slice images were exported in PNG format (grayscale, 8-bit) using the DICOM viewer Miele-LXIV. The image size was 128 × 128 pixels, with a pixel size of 3.29 mm. Next, the peripheral areas of the brain were removed due to their lack of relevant information. The central 64 × 64 pixels of each image were cropped using the image processing library Pillow. The 1035 extracted images were then divided by acquisition time into training (120 cases, 600 images), validation (37 cases, 185 images), and test (50 cases, 250 images) datasets. For image interpretation, the color depth was changed to 16-bit using the image processing software ImageJ (version 1.53t, National Institutes of Health, Bethesda, MD, USA), and the “Cool” color map was applied to generate color images which were then output in PNG format. The default window and level values were used without adjustment. For each case, only three slices were used for interpretation: the slice with the highest maximum count number and the slices immediately before and after.

### Deep-learning model

The models used are U-Net, V-Net, U-Net +  + , R2U-Net, Attention U-Net, and TransUNET with Mean Squared Error** (**MSE) for “loss function” and 5 slices for “multi-slice input”. For reference, we also compare the results of applying image processing techniques, Gaussian filtering and bilateral filtering. Five patterns of U-Net with 1, 2, 3, 4, and 5 layers are prepared to compare the accuracy depending on the number of layers. Other U-Net-related models have 4 layers. The number of filters for 1-layer U-Net is 16, for 2-layer U-Net is 16, 32, for 3-layer U-Net is 16, 32, 64, for 4-layer U-Net is 16, 32, 64, 128, and for 5-layer U-Net is 16, 32, 64, 128, 256. The parameters during learning are 200 epochs, a batch size of 8, and the Adam optimizer. Similar to a previous report [15], the learning rate is initially set to 1.0 × 10^–3^ and decreased to 1.0 × 10^–5^ after 150 epochs. The complete hyper-parameter settings for all comparison networks (V-Net, U-Net +  + , R2U-Net, Attention U-Net and TransUNet) are detailed in Supplementary Figure S1.In this experiment, in addition to quantitative evaluation, visual evaluation is also performed.

Furthermore, an additional experiment is conducted to confirm evaluator reliability. As intra-evaluator reliability, to measure the reproducibility and consistency of the image reading results, only the U-Net (L4) reading files are duplicated after 4 weeks, and a second visual evaluation is performed. Subsequently, inter-evaluator reliability is confirmed from the results of the visual evaluation.

## Evaluation Method

### Quantitative Evaluation

Two evaluation metrics, PSNR (Peak Signal to Noise Ratio) and SSIM (Structural Similarity), were used. These metrics are currently the de facto standard for evaluating noise reduction tasks. In this experiment, the ground truth data is a 25-min captured image of the same slice. The mean and standard deviation for each metric were calculated across the test data. Furthermore, a Friedman test was conducted to confirm the overall differences between all models, followed by a Dunn test (Holm correction) to compare U-Net (L4) with other methods.

### Visual assessment

Three nuclear medicine physicians independently graded the striatal uptake into four categories and compared the diagnostic scores between the short-time acquisition virtual images and the standard images. One score was assigned for each side (left and right) per case. To accommodate hemisphere-specific (unilateral) grading of the striatum, we adapted the pattern-based visual scale originally reported by Kahraman et al. [16]. The modified four-point system was defined as follows: burst striatum = 1, egg shape = 2, eagle wing = 3, and normal = 4 [16].

The reading data is shared with the nuclear medicine physicians as image files, separated for each model. Each file contains colorized and magnified images of three representative slices (as defined in the preprocessing section) for 50 test cases. Nuclear medicine physicians determine a single diagnostic score for each side (i.e., a per-patient, per-striatum score) based on reviewing the three representative images. Model names and case numbers are blinded and randomized. For the 25-min captured image (original image), a separate consensus reading is performed by three nuclear medicine physicians, which serves as the gold standard.

After the reading, the results are compiled, and the quadratic weighted kappa coefficient with the gold standard is calculated for each. This represents the degree of agreement with the 25-min captured image evaluation.

In the additional experiment, intra-evaluator reliability is assessed by calculating the Intraclass Correlation Coefficient between the first and second U-Net diagnostic scores for each nuclear medicine physician. Inter-evaluator reliability is assessed by calculating the Intraclass Correlation Coefficient using all diagnostic scores (original image (5 min), U-Net, V-Net, U-Net +  + , R2U-Net, Attention U-Net, TransUNET) from each nuclear medicine physician.

## Statistical analysis

### Quantitative image quality

For every test slice (n = 250) the PSNR and SSIM were calculated and reported as mean ± standard deviation. Because the 5-min acquisition and each deep-learning reconstruction were obtained from the same slice, overall differences among methods were first assessed with the Friedman test. When this test was significant (two-sided p < 0.05), pair-wise post-hoc comparisons between the reference model U-Net(L₄) and each alternative method were conducted using Dunn’s test with Holm adjustment for multiple comparisons.

### Visual assessment

Three nuclear-medicine physicians independently scored 50 test cases (100 striata) on a four-point scale, using the 25-min consensus image as the gold standard. Agreement between each reconstruction and the reference was quantified with quadratically weighted Cohen’s κ. Reader consistency was further examined by (i) re-reading the U-Net(L₄) images after a 4-week wash-out to obtain intra-rater reliability (intraclass correlation coefficient, ICC) and (ii) calculating inter-rater ICC across the three observers.

All statistical tests were two-sided; p < 0.05 after multiplicity correction was considered significant.

## Results

The gold standard ratings assigned to the original images of the 100 striata from the 50 cases analyzed in the visual interpretation experiment were distributed as follows: score 4, n = 26; score 3, n = 28; score 2, n = 29; and score 1, n = 17. Across the 100 striata, all deep-learning reconstructions significantly improved objective image quality over the 5-min raw acquisitions (Friedman test *p* < 0.01 for both PSNR and SSIM; Table 2 and 3). The four-layer U-Net achieved the highest scores, with a mean PSNR of 32.7 ± 1.7 dB and a mean SSIM of 0.842 ± 0.069, surpassing the short-time images (30.8 ± 0.7 dB and 0.708 ± 0.081, respectively). This corresponds to a gain of roughly 1.8 dB (≈ 6%) in PSNR and 0.13 (≈ 19%) in SSIM relative to the 5-min baseline. Other CNN variants (U-Net L1–L3, L5, V-Net, U-Net +  + , R2U-Net, Attention U-Net, and TransUNet) also produced PSNR values above 32 dB and SSIM values above 0.82, but none showed a statistically significant advantage over U-Net L4 after Holm-corrected Dunn tests (*p* > 0.05). These findings indicate that deep-learning reconstruction—particularly the 4-layer U-Net—can recover image quality nearly indistinguishable from the 25-min reference while starting from a five-min acquisition.

**Table 2:** Peak signal-to-noise ratio

	Mean ± SD	*P*-value (vs. U-Net(L_4_))
Short time image(5 min)	30.837 ± 0.747	< 0.01
Gaussian filtering	30.980 ± 0.836	< 0.01
Bilateral filtering	31.078 ± 0.935	< 0.01
U-Net(L_1_)	32.294 ± 1.339	1.0
U-Net(L_2_)	32.390 ± 1.494	1.0
U-Net(L_3_)	32.327 ± 1.388	1.0
U-Net(L_4_)	32.683 ± 1.678	
U-Net(L_5_)	32.610 ± 1.685	1.0
V-Net	32,371 ± 1.441	1.0
U-Net + +	32.043 ± 1.204	1.0
R2U-Net	32.490 ± 1.542	1.0
Attention U-Net	32.529 ± 1.582	1.0
TransUNET	32.517 ± 1.567	1.0

*U-Net* U-Net (encoder–decoder CNN), *U-Net* +  + Nested U-Net, *V-Net* V-Net (3-D CNN), *R2U-Net* Recurrent residual U-Net, *Attention U-Net* Attention-gated U-Net, *TransUNet* Transformer-based U-Net

**Table 3:** Structural similarity index

	Mean ± SD	*P*-value (vs. U-Net(L_4_))
Short time image (5 min)	0.708 ± 0.081	< 0.01
Gaussian filtering	0.747 ± 0.077	< 0.01
Bilateral filtering	0.773 ± 0.076	< 0.01
U-Net(L_1_)	0.822 ± 0.056	1.0
U-Net(L_2_)	0.829 ± 0.059	1.0
U-Net(L_3_)	0.828 ± 0.064	1.0
U-Net(L_4_)	0.842 ± 0.069	
U-Net(L_5_)	0.837 ± 0.066	1.0
V-Net	0.825 ± 0.066	1.0
U-Net + +	0.813 ± 0.067	0.294
R2U-Net	0.833 ± 0.067	1.0
Attention U-Net	0.840 ± 0.070	0.713
TransUNET	0.831 ± 0.071	1.0

*U-Net* U-Net (encoder–decoder CNN), *U-Net* +  + Nested U-Net, *V-Net* V-Net (3-D CNN), *R2U-Net* Recurrent residual U-Net, *Attention U-Net* Attention-gated U-Net, *TransUNet* Transformer-based U-Net

### Visual evaluation

Visual interpretation of the reconstructed images was performed independently by three board-certified nuclear-medicine physicians. Agreement with the 25-min reference images was quantified by quadratic weighted κ (Table 4).

- *Baseline 5-min acquisitions* The raw 5-min images showed only fair concordance with the reference standard, yielding κ values of 0.37, 0.29, and 0.41 for Readers A, B, and C, respectively.
- *U-Net (four-layer) reconstructions* Application of the four-layer U-Net markedly improved concordance, increasing the κ coefficients to 0.62, 0.70, and 0.67 for Readers A–C. These values fall within the substantial agreement category of Landis–Koch.
- *Alternative CNN architectures* Other tested networks (e.g., U-Net +  + , V-Net, Attention U-Net) produced κ values ranging from 0.64 to 0.73, likewise indicating substantial agreement. Formal pairwise statistical comparisons between models were not undertaken; the κ estimates are provided descriptively to illustrate the magnitude of improvement over the short-time baseline.

**Table 4:** Weighted kappa coefficient for visual assessment

	Reader A	Reader B	Reader C	Average
Short time image(5 min)	0.370	0.292	0.413	0.358
U-Net(L_4_)	0.616	0.696	0.670	0.661
V-Net	0.643	0.732	0.652	0.676
U-Net + +	0.630	0.647	0.422	0.566
R2U-Net	0.537	0.625	0.626	0.594
Attention U-Net	0.630	0.679	0.699	0.669
TransUNET	0.579	0.716	0.578	0.624

*U-Net* U-Net (encoder–decoder CNN), *U-Net* +  + Nested U-Net, *V-Net* V-Net (3-D CNN), *R2U-Net* Recurrent residual U-Net, *Attention U-Net* Attention-gated U-Net, *TransUNet* Transformer-based U-Net

Reproducibility analyses confirmed excellent observer consistency:

- *Intra-rater reliability* After a four-week wash-out period, re-evaluation of 50 randomly duplicated cases yielded intra-observer ICC values of 0.84 (Reader A), 0.88 (Reader B), and 0.93 (Reader C) (Table 5).
- *Inter-rater reliability* Across the entire cohort of 100 striata, inter-observer ICC values were 0.74 (A vs B), 0.75 (A vs C), and 0.73 (B vs C), all indicative of substantial agreement (Table 5).

**Table 5:** Intra class correlation coefficient

Intra-rater reliability		Inter-rater reliability	
Reader A	0.838	Reader A–Reader B	0.737
Reader B	0.879	Reader A–Reader C	0.74*
Reader C	0.928	Reader B–Reader C	0.728
Average	0.882	Average	0.73*

Collectively, these results demonstrate that deep-learning reconstruction—particularly the four-layer U-Net—substantially narrows the perceptual gap between 5-min and 25-min ^123^I-Ioflupane SPECT acquisitions while maintaining high intra- and inter-observer reliability.

**Fig. 2:**
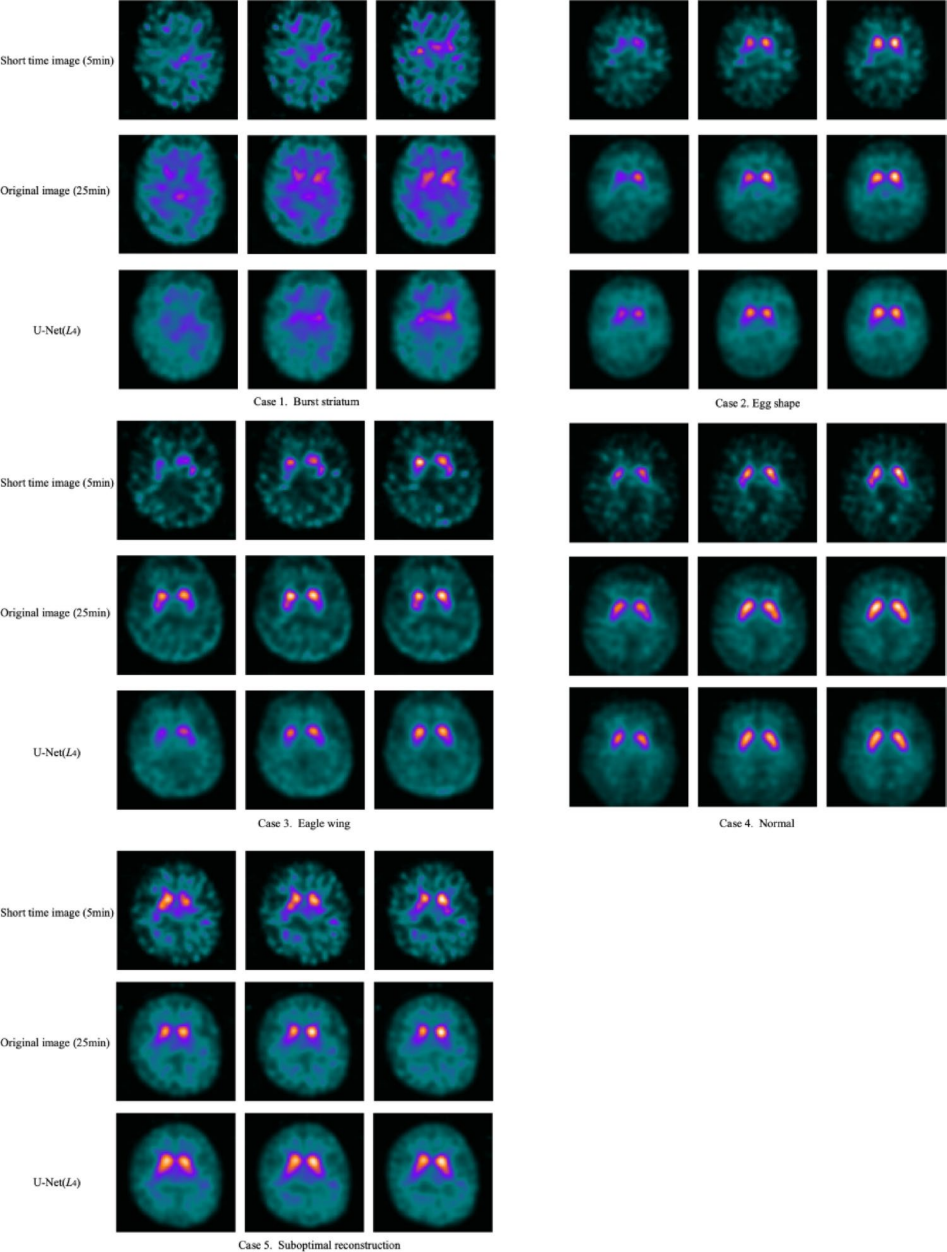
Representative cases by visual assessment score: Comparison of 25-min reference images and U-Net(*L*4) reconstructions. Case 1: Burst striatum (score 1). In both the original 25-min image and the U-Net(*L*4) reconstruction, striatal uptake was approximately equivalent to the background. This score 1 finding was accurately reproduced bilaterally by the U-Net(*L*4) model. Case 2: Egg shape (score 2). The original 25-min image demonstrated severely reduced uptake in the posterior striatum, with residual egg-shaped uptake visible in the caudate nucleus. The U-Net(*L*4) reconstruction successfully reproduced this score 2 pattern bilaterally. Case 3: Eagle wing (score 3). The original 25-min image showed mildly reduced uptake in the posterior striatum. This score 3 pattern was also accurately reproduced bilaterally by the U-Net(*L*4) model. Case 4: Normal (score 4). The original 25-min image demonstrated normal striatal uptake. The U-Net(*L*4) reconstruction reproduced this normal score 4 pattern bilaterally. Case 5: Suboptimal reconstruction. The original 25-min image was rated as score 2 bilaterally. In contrast, the U-Net(*L*4) reconstruction was rated as score 3 or 4, suggesting potential over-correction by the deep-learning model

**Fig. 3:**
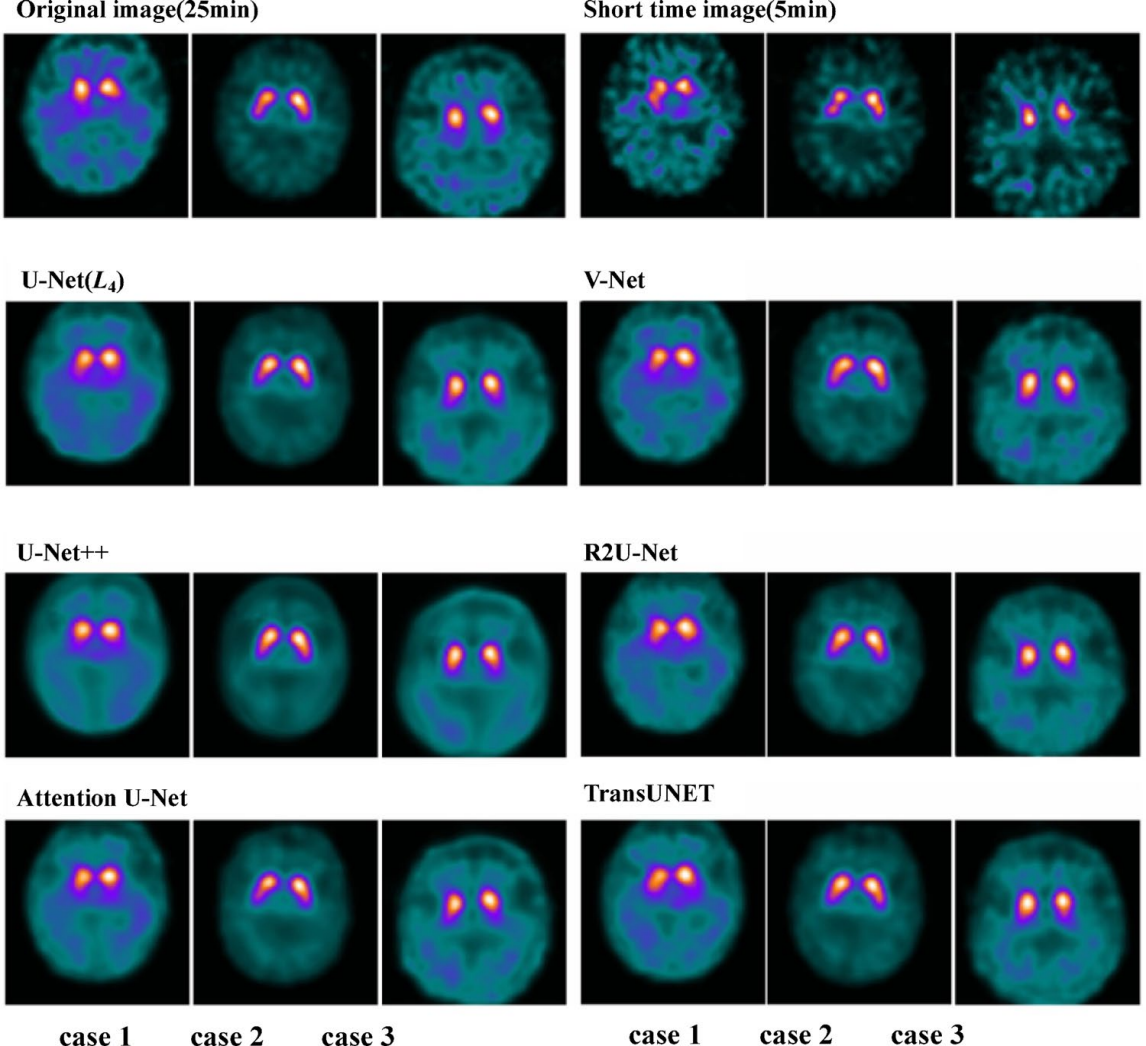
Qualitative performance of deep-learning reconstructions for short-time ^123^I-ioflupane SPECT. For three representative patients (columns), axial slices are shown for the 25-min reference image, the 5-min short-time image, and six deep-learning reconstructions (U-Net [*L*₄], V-Net, U-Net +  + , R2U-Net, Attention U-Net, and TransUNET). All panels share an identical color scale and intensity window. Deep-learning methods markedly sharpen striatal borders and reduce background noise when compared with the 5-min baseline, with the four-layer U-Net appearing most similar to the 25-min reference. *U-Net* U-Net (encoder–decoder CNN), *U-Net* +  + Nested U-Net, *V-Net* V-Net (3-D CNN), *R2U-Net* Recurrent residual U-Net, *Attention U-Net* Attention-gated U-Net, *TransUNet* Transformer-based U-Net

Figure 2 provides representative examples based on the 4-point visual assessment scale, comparing the 25-min reference, the 5-min short-time image, and the U-Net(L4) reconstruction. Cases 1–4 (scores 1–4) demonstrate that the U-Net(L4) model successfully reproduced the diagnostic patterns, showing a close qualitative match to the 25-min standard. Case 5 illustrates a suboptimal case where the U-Net(L4) model appeared to over-correct the image, aligning with the “over-smoothed” phenomenon discussed in the limitations. Figure 3 presents three representative cases comparing the 25-min reference image, the 5-min short-time acquisition, and the corresponding deep-learning reconstructions from all six architectures. Relative to the short-time baseline, all networks visibly restored striatal contrast and suppressed background noise, with the four-layer U-Net (U-Net(L4)) showing the closest qualitative match to the 25-min standard.

## Discussion

This study demonstrates the feasibility of reconstructing diagnostic-quality ^123^I-ioflupane SPECT images from a 5-min acquisition, which is only one-fifth of the conventional scan time. Using a 4-layer U-Net, we achieved high-fidelity images that closely replicate the 25-min standard, as evidenced by strong quantitative metrics (significant increases in PSNR and SSIM relative to the unenhanced 5-min images) and excellent qualitative agreement with reference scans. In fact, the U-Net–enhanced short acquisitions yielded a weighted κ ≈ 0.7 in blinded visual assessments, indicating substantial agreement with the standard 25-min images. To our knowledge, this work represents the first successful application of deep learning to drastically shorten imaging time in dopamine transporter SPECT. The ability to obtain reliable ^123^I-ioflupane scans in 5 min carries important clinical benefits: it minimizes patient motion and discomfort, reduces the likelihood of patients abandoning the exam, and improves scanner throughput and cost-effectiveness. In short, our results show that advanced image enhancement can overcome low counts and preserve diagnostic content even at 80% time reduction, which is a novel and impactful finding in the field of nuclear medicine.

Our findings are in line with a growing body of research applying deep learning to accelerate SPECT and PET imaging. In the SPECT realm, most prior studies have focused on myocardial perfusion imaging. For example, Shiri et al*.* [9] used a deep residual CNN to estimate full-time-equivalent cardiac SPECT images from half-time acquisitions, and reported that perfusion defect detection remained nearly as accurate as with the original full-time scans [9]. Similarly, Aghakhan-Olia et al*.* (2022) showed that a deep learning denoising model could maintain quantitative accuracy and clinical interpretability in SPECT myocardial perfusion images even at substantially reduced dose levels [17]. Ramon et al*.* [18] also demonstrated improved diagnostic accuracy in low-dose cardiac SPECT by using a convolutional denoising network [18]. Beyond the heart, deep learning has been applied to other SPECT studies: Qi et al*.* [19] recently reported that a CNN could enhance whole-body bone SPECT scans acquired in only 1/7^th of the normal time (3 min vs. 20 min), yielding images with comparable lesion detectability and reader confidence to standard scans [19]. In that prospective study, the ultrafast CNN-enhanced SPECT showed no significant difference in diagnostic performance compared to the full acquisition, with high inter-observer agreement (κ ≈ 0.73–0.82) and significantly improved image quality metrics (SSIM and PSNR) over the raw low-count images. These peer-reviewed examples underscore that deep learning can compensate for reduced counts or shortened acquisitions in nuclear medicine, which concurs with the success we observe in accelerating brain SPECT. Notably, however, before our work there were no published studies applying such techniques to brain dopaminergic SPECT imaging – a gap that our study begins to fill.

Deep learning has likewise shown promise in fast or low-dose PET imaging, and our results are consistent with those trends. For instance, in whole-body FDG PET, Chaudhari et al*.* [20] achieved a four-fold count reduction using a deep learning enhancement model without compromising diagnostic quality [20]. In their multicenter evaluation, 25%-count PET images enhanced by a CNN were rated non-inferior to standard scans, with lesion detection sensitivity > 94% and an inter-method κ of ~ 0.85 (comparable to intra-reader variability). Other groups have explored more complex architectures such as adversarial networks to improve low-dose PET. For example, Wang et al*.* [21] introduced a 3D cGAN model that produced high-quality PET images from severely undersampled data [21]. These studies confirm that advanced networks can recover fine image details and quantitative accuracy even when PET dose or scan time is dramatically reduced. The success of our comparatively simple U-Net on short-acquisition SPECT aligns with the broader evidence that deep learning methods can effectively denoise and correct low-count images, enabling substantial reductions in radiotracer dose or acquisition duration across modalities.

We chose an image-enhancement approach over an end-to-end (E2E) diagnostic classification model for strategic reasons related to clinical safety and workflow. While modern E2E models have improved interpretability through techniques such as attention maps [22, 23], they are typically trained to perform specific binary or multi-class classification tasks (e.g., differentiating Parkinson’s disease from healthy controls). Consequently, such models risk overlooking incidental findings or complex pathologies that fall outside their specific training labels but are clinically critical.

In contrast, our reconstruction approach acts as an assistive tool that provides a high-fidelity visual image, preserving the “human-in-the-loop” workflow. This allows nuclear medicine physicians to utilize their full expertise to verify tracer distribution and assess the entire scan for unexpected abnormalities. Furthermore, generating a standard diagnostic image ensures seamless integration into existing Picture Archiving and Communication Systems (PACS) and aligns with current medical standards where the visual image, interpreted by a physician, serves as the primary basis for the medical record and legal liability.

One important aspect of our approach is its architectural simplicity. We found that a standard 4-layer U-Net was sufficient to outperform or match not only a deeper 5-layer U-Net, but also several more elaborate network variants (including U-Net +  + , R2U-Net, Attention U-Net, and TransUNet) in both quantitative and qualitative evaluations. Increasing the network depth beyond 4 layers did not yield any appreciable improvement in PSNR/SSIM or visual. This outcome may reflect the nature of our data: the input slices were relatively small (64 × 64 pixels) and focused on a consistent anatomical region (the striata in mid-brain), which limits complexity and variability. A deeper or more complex model could be prone to overfitting under these circumstances, whereas the 4-layer U-Net captured the essential features needed to map 5-min images to their 25-min counterparts. From a practical standpoint, the success of a shallow network is encouraging – the model has fewer parameters and lower computational demand, making it easier to train and deploy in clinical settings. In our study, even this compact U-Net produced markedly cleaner images (sharpening striatal borders and suppressing noise) that were almost indistinguishable from the full-count images (Fig. 2), which speaks to the effectiveness of the U-Net architecture for this task.

### Limitations and future directions

Despite the promising results, this study has several limitations that suggest avenues for future research. First, our analysis was retrospective and single-center, using data from one hospital and one SPECT/CT system. Differences in imaging protocols or scanner hardware could affect the model’s performance; therefore, multi-center validation is needed. Future work should include external datasets from other institutions to ensure the U-Net generalizes well and to address inter-site variations in SPECT images (e.g. due to camera calibrations or collimators). Developing strategies for cross-center harmonization using deep learning is an exciting prospect to standardize image quality and quantitative metrics across different scanners.

Second, our network was trained on 2D slices centered on the striatum after rigid preprocessing. While this region-of-interest approach optimized our model for the critical diagnostic content (dopamine transporter uptake in the basal ganglia), it may not account for abnormalities outside the cropped area. In the future, incorporating 3D networks or processing the entire brain volume could allow detection of off-target tracer uptake or incidental findings, albeit at the cost of needing more training data.

Third, the current model focused on a static post-injection time point of a specific tracer. The underlying concept, however, could be extended to other radiotracers and imaging contexts. For example, applying similar U-Net based enhancement to brain perfusion SPECT (e.g. ^99m^Tc-ECD) or to dynamic SPECT acquisitions might be fruitful. We chose ^123^I-ioflupane as a starting point in part because of its stable biodistribution after the uptake phase, which theoretically makes rapid acquisition feasible; other tracers with time-varying distribution might require more sophisticated temporal modeling or gating.

Fourth, our study design compared only the 5-min (20%) acquisition with the 25-min standard, representing an 80% time reduction. We did not investigate intermediate durations, such as a 50% 'half-time’ acquisition (e.g., ~ 12.5 min), which may be more readily adopted in some clinical settings. Our model was trained specifically for the 5-to-25 min task and would not directly generalize to other input durations. While we hypothesize that a model trained on a half-time scan would produce excellent, perhaps superior, results due to the higher quality input, this remains to be tested. Evaluating the performance scaling at different intermediate time points is a key area for future work.

A further limitation involves the visual assessment protocol. The four-point scale we adopted was used as a standardized method to quantify *agreement* (weighted κ), not to encompass the full spectrum of clinical diagnostic interpretation. Clinical diagnosis relies on more complex factors than these four patterns, critically including the assessment of relative uptake intensity and laterality (asymmetry). Our study required physicians to score the left and right striatum independently. While this design allowed us to measure the agreement of uptake scores for each hemisphere individually, it did not include a formal, separate evaluation of asymmetry. Therefore, the clinical interpretation of these complex diagnostic features was not explicitly part of our visual scoring task. Future studies should be designed to specifically assess how these DL-enhanced images impact the clinical interpretation of the full range of diagnostic features, including striatal asymmetry.

Furthermore, our validation focused on global image quality metrics (PSNR, SSIM) and qualitative visual assessment. We did not evaluate the impact of our DL reconstruction on specific quantitative indices, such as the specific binding ratio, asymmetry index, or caudate-to-putamen ratio, which are routinely used in clinical practice. This is a significant limitation. Future validation is essential to determine whether DL-enhanced images maintain the accuracy and reliability of these objective measurements, or if the reconstruction process introduces any bias to these quantitative parameters.

Most importantly, the focus of our validation was on image fidelity (PSNR, SSIM, and visual agreement) rather than diagnostic accuracy. Although our dataset included a range of parkinsonian syndromes (as detailed in Table 1), we did not evaluate whether the DL-reconstructed images compromised the specific ability to differentiate these diseases (e.g., PD vs. PSP, or DLB vs. ET). A future, prospective study focused on diagnostic accuracy using confirmed clinical diagnoses as the ground truth is essential before this technique can be safely adopted.

Another limitation is the small training dataset (600 images from 120 cases), which, while augmented by similarity of slices, is modest by deep learning standards. A larger dataset could improve the model’s noise-generalization properties and prevent any subtle artifacts. We did observe in a few cases that the network-produced images appeared over-smoothed or visually clearer than the 25-min reference images, as reflected by readers occasionally giving higher scores to the synthetic images than to the standard images. This phenomenon suggests the model may learn to suppress noise so aggressively that it alters texture – an issue that warrants further investigation to ensure no diagnostic information is inadvertently erased. Using next-generation architectures like diffusion models or incorporating physics-based constraints might help achieve even higher fidelity reconstructions. In ongoing and future studies, we plan to increase the training set size and evaluate such advanced models to see if they can push image quality closer to the theoretical limit of a full acquisition.

Finally, a prospective clinical trial will be essential to confirm that radiologists and referring physicians can use these AI-enhanced 5-min SPECT images safely in practice. By addressing these issues, we hope to move this approach toward clinical translation and also explore its application to other brain SPECT examinations that could benefit from shorter scan times.

## Conclusion

This study provides the first evidence that a 5-min ^123^I-ioflupane SPECT acquisition, processed with a compact four-layer U-Net, can yield images that are quantitatively and qualitatively indistinguishable from the conventional 25-min protocol. The network increased PSNR and SSIM by approximately 6% and 19%, respectively, while achieving substantial inter-observer agreement (weighted κ ≈ 0.7) against the reference standard. By demonstrating reliable dopamine-transporter imaging at one-fifth of the usual scan time, our work offers tangible clinical benefits—shorter patient immobilisation, reduced motion artefacts, and markedly improved scanner throughput—without sacrificing diagnostic confidence.

Equally important, the architectural simplicity of the four-layer U-Net underscores that sophisticated, resource-intensive models are not strictly necessary to achieve state-of-the-art performance in low-count SPECT reconstruction. This lowers the barrier to real-world implementation and facilitates deployment on standard clinical hardware.

Future research should validate these findings across multiple centres and camera vendors, explore 3-D or dynamic extensions to accommodate other tracers and protocols, and evaluate next-generation architectures (e.g., diffusion or physics-informed models) for further gains. A prospective clinical trial will be essential to confirm safety and efficacy in routine practice. Nonetheless, the present results establish a new benchmark for accelerated dopamine-transporter SPECT and lay the groundwork for broader applications of deep learning–based time reduction in nuclear medicine.

## Supplementary Information

Below is the link to the electronic supplementary material.

Supplementary file1 (DOCX 147 kb)

## Abbreviations

CNN: Convolutional neural network
cGAN: Conditional generative adversarial network
DDSRCNN: Deep Denoising Super-Resolution Convolutional Neural Network
123I: Iodine-123
FP-CIT: N-(3-fluoropropyl) -2*β*-carbomethoxy-3*β*-(4-iodophenyl) nortropane
DL: Deep learning
ICC: Intraclass correlation coefficient
PSNR: Peak signal-to-noise ratio
SSIM: Structural similarity index measure
SPECT: Single-photon emission computed tomography
U-Net: U-Net (encoder–decoder CNN)
U-Net + +: Nested U-Net
V-Net: V-Net (3-D CNN)
R2U-Net: Recurrent residual U-Net
Attention U-Net: Attention-gated U-Net
TransUNet: Transformer-based U-Net
ROI: Region of interest
