# Supplementary material for: Accelerating imaging: deep learning for enhanced 123I-ioflupane SPECT efficiency
Source: Jpn J Radiol. 2025 Dec 18;44(5):905–15. doi: 10.1007/s11604-025-01933-z (PMC13144268; doi:10.1007/s11604-025-01933-z)
Supplement: Supplementary file 1 — Supplementary file1 (DOCX 147 kb) [file 11604_2025_1933_MOESM1_ESM.docx]

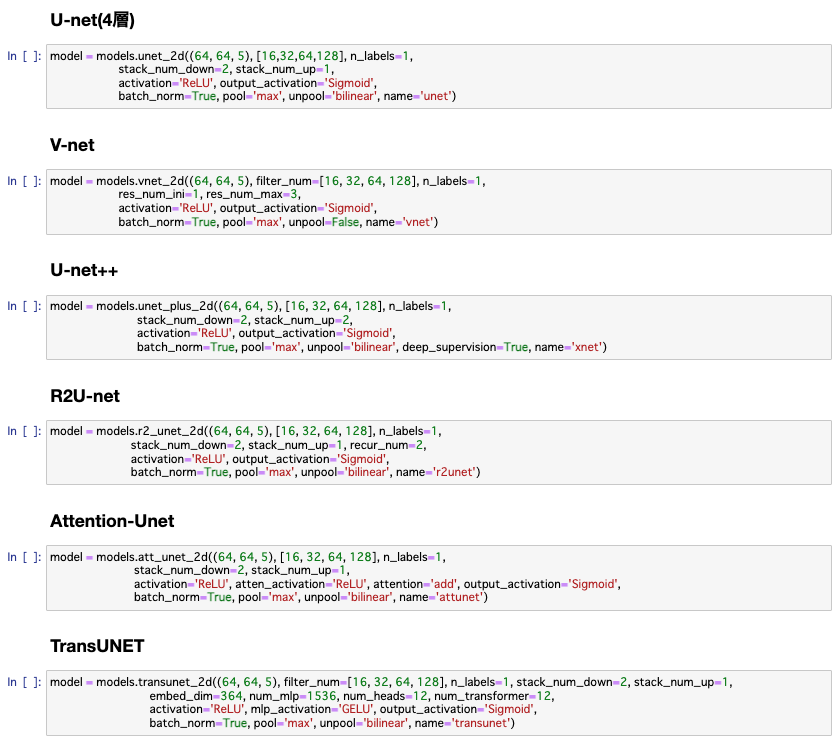


**Supplementary Figure S1: Hyper-parameter configurations for all comparison deep-learning models.**
